# Radiographic features of congenital thumb duplication type C2 of Wu et al. classification: new subtypes and surgical strategies

**DOI:** 10.3389/fped.2023.1286662

**Published:** 2024-01-12

**Authors:** JianPing Wu, WeiZhe Shi, Hai Zhao, JingChun Li, YiQiang Li, Kai Hong, Zhe Yuan, MingWei Zhu, YuanZhong Liu, Federico Canavese, HongWen Xu

**Affiliations:** ^1^ Department of Pediatric Orthopaedics, Guangzhou Women and Children’s Medical Center, Guangdong Provincial Clinical Research Center for Child Health, Guangzhou, China; ^2^ Department of Pediatric Orthopedic, Chenzhou No.1 People’s Hospital, Chenzhou, China; ^3^ Department of Pediatric Orthopedic Surgery, Lille University Center and Faculty of Medicine Henri Warenbourg, Jeanne de Flandre Hospital, Lille, France

**Keywords:** congenital thumb duplication, Wu et al. classification, anatomy, interphalangeal joint alignment, surgery

## Abstract

**Objective:**

This study aimed (i) to evaluate the radiographic characteristics of patients with congenital thumb duplication (CTD) type C2 according to the classification of Wu et al., (ii) to describe the various subtypes of type C2 CTD, and (iii) to propose a classification system that allows the identification of different surgical strategies based on the radiographic anatomy of this specific subtype of duplication.

**Methods:**

We retrospectively reviewed 92 patients (92 thumbs) with type C2 CTD according to the Wu et al. classification in our institution between August 2015 and April 2021. All CTDs were classified according to the interphalangeal joint alignment of the main thumb at the posteroanterior radiograph of the thumb before operation: type I (no deviation), type II (ulnar deviation), and type III (radial deviation).

**Results:**

All CTDs (*n* = 92) could be classified according to the proposed classification system: 76 (82.6%) were type I, 10 (10.9%) were type II, and six were type III (6.5%). According to the Kim system of subtype classification, there were 55 (59.8%) type 1, 24 (26.1%) type 2, and 13 (14.1%) type 3 cases.

**Conclusions:**

The suggested classification completes the Wu et al. system and has the potential to guide surgical treatment in children with type C2 CTD.

**Level of evidence:**

III.

## Introduction

Congenital thumb duplication (CTD) is one of the most common hand deformities in children (1–3). The local anatomy can be particularly complex and can include bony abnormalities and abnormal localization of tendons and thenar muscle (4–6). Therefore, simple excision of the supernumerary thumb may be insufficient, and adequate bony and soft tissue reconstruction is required (7–9).

Wassel–Flatt type III CTD, in which the duplicated thumb has a bony attachment at the level of the proximal phalanx, is a rare malformation as it accounts for between 3% and 10% of all CTDs (2, 6, 7). Furthermore, few reports on CTD III alone, mostly with a small number of patients, are available in the literature (2, 8–11). For example, Horii et al. described 11 cases of type III CTD treated with radial thumb excision and size augmentation and concluded that the technique provides a functionally good thumb (12). Kim et al. divided 32 cases of type III CTD into three subtypes (1–3) based on the alignment of the duplicated thumb and evaluated the applied surgical procedures and outcomes. However, these works have not described the special cases of type III CTD in which the supernumerary thumb is connected by bone tissue to the proximal phalanx and which do not correspond to the classic bifurcated phalanx (2). No study to date has specifically evaluated the radiographic features of proximal phalanx deformity in type III CTD.

To overcome some of these problems, Wu et al. introduced a modified radiographic classification for CTD that allows the classification of all forms of CTD with excellent inter- and intra-observer reliability (10). Specifically, type C2 of the Wu et al. classification includes all types of type III CTD described in the works of Horii et al. and Kim et al. In particular, it allows the various subtypes to be identified on the basis of bony connection, regardless of the presence of a typical proximal bifurcated phalanx (1, 2, 10–12).

The objectives of this study were (i) to evaluate the radiographic characteristics of patients with CTD type C2 according to the classification of Wu et al. who were seen at our center between August 2015 and April 2021, (ii) to describe the various subtypes of type C2 CTD, and (iii) to propose a classification system that allows the identification of different surgical strategies based on the radiographic anatomy of this specific subtype of duplication.

## Materials and methods

After securing approval from the Institutional Review Board (IRB) of our hospital (316B01) and obtaining informed consent from the parents or legal guardians of the study participants, we retrospectively reviewed the medical records of 92 children (*n* = 92 thumbs) who were diagnosed with type C2 CTD according to the Wu et al. classification between August 2015 and April 2021 at our institution (10).

The inclusion criteria were as follows: (1) confirmed diagnosis of type C2 CTD according to the Wu et al. classification, (2) complete clinical and radiological data, and (3) treatment exclusively performed at our institution.

Patients with incomplete clinical and radiological data, CTD other than type C2 according to the Wu et al. classification, and those managed elsewhere were excluded.

### C2 CTD subtypes

The authors proposed a new system for classifying the various subtypes of C2 CTD from the Wu et al. classification.

The identification of the various subtypes is essentially based on the alignment of the interphalangeal (IP) joint of the main thumb rather than that of the supernumerary thumb.

**Figure 1:**
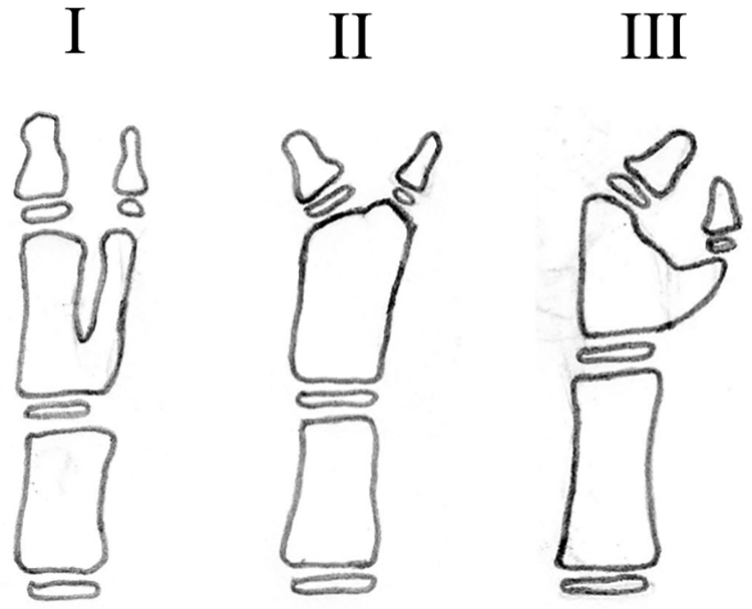
Subtypes of type C2 CTD based on the Wu et al. classification (10). Type I: good IP alignment of the main thumb without radial or ulnar deviation. Type II: ulnar deviation of the IP joint of the main thumb. Type III: radial deviation of the IP joint of the main thumb.

Type I identifies cases in which there is no axial deviation; the alignment of the IP joint of the main thumb is good, with no radial or ulnar deviation. Type II identifies cases where there is ulnar deviation of the main thumb; the IP joint of the first thumb has deviated to the ulnar side. Type III identifies cases with radial deviation of the main thumb; the alignment of the IP joint of the main thumb has radial deviation (Figure 1).

### Surgical strategies

The classification system of CTD type C2 subtypes is based on the radiographic and clinical anatomy of the duplications and is related to the surgical procedures that can be implemented.

**Figure 2:**
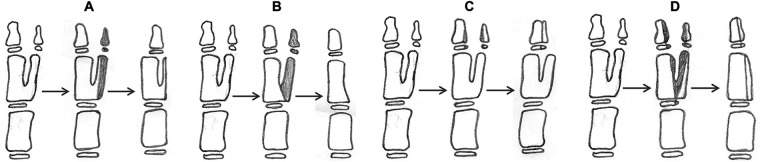
Surgical reconstruction of type I duplication. (**A**) resection of the distal phalanx and partial resection of the proximal phalanx; reconstruction of the collateral ligament is needed. (**B**) Resection of proximal and distal phalanx with reconstruction of the collateral ligament and thenar muscle. (**C**) Bilhaut–Cloquet procedure at the distal phalanx only. (**D**) Bilhaut–Cloquet procedure at the proximal and distal phalanx. (**C**) and (**D**) require soft tissue reconstruction with or without nail bed reconstruction.

For type I forms with good alignment of the main thumb and IP, the surgical option is excision of the supernumerary thumb and reconstruction of the soft tissues (tendons, thenar muscle, flexor or extensor longus of the thumb) (Figures 2A,B). Another option is Bilhaut–Cloquet surgery, which includes reconstruction of the distal phalanx alone (Figure 2C) or both the distal and proximal phalanges (Figure 2D) with soft tissue reconstruction, with or without nail bed reconstruction (8, 12–14).

**Figure 3:**
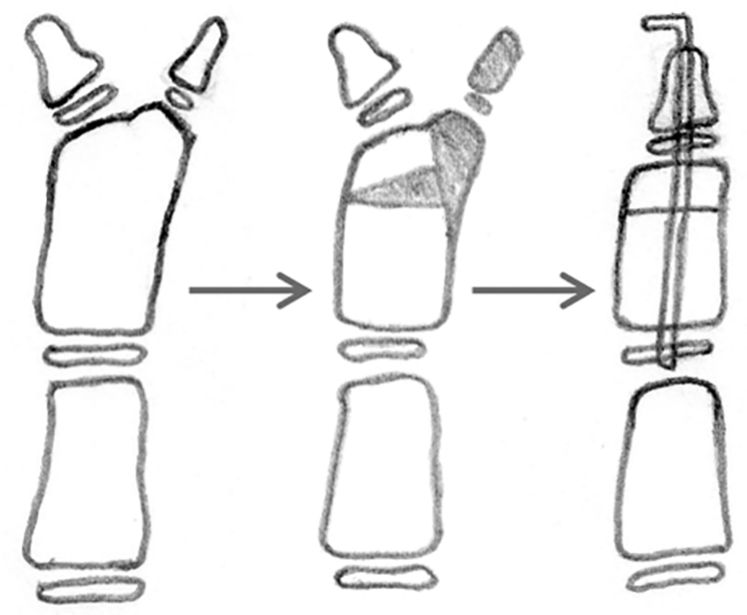
Reconstruction of type II duplication with ulnar deviation; the extra thumb is excised, and corrective osteotomy of the proximal phalanx and ligament/periosteal sleeve reconstruction is performed.

For type II duplications, with ulnar deviation of the main thumb and IP, the supernumerary thumb is excised, and an osteotomy of the proximal phalanx and reconstruction of the periosteal ligament/sleeve must be performed (Figure 3).

**Figure 4:**
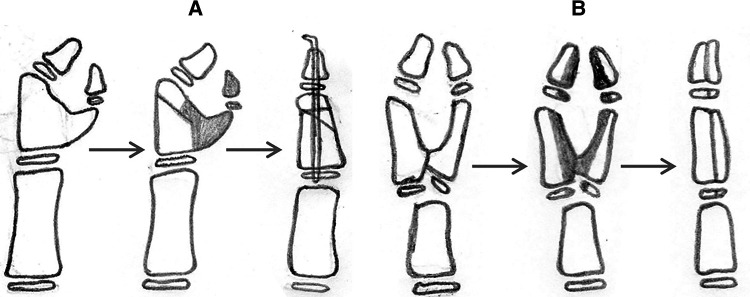
Reconstruction of type III duplication with radial deviation. (**A**) The extra thumb is excised, and corrective osteotomy of the proximal phalanx, a reconstruction of the ligament/periosteal sleeve, and a reconstruction of the thenar muscle or flexor tendon are performed. (**B**) Bilhaut–Cloquet procedure and soft tissue reconstruction with or without nail bed reconstruction.

For type III CTDs, with radial deviation of the main thumb and IP, the supernumerary thumb is excised and an osteotomy of the proximal phalanx, a reconstruction of the periosteal ligament/sleeve, and a reconstruction of the thenar muscle or flexor tendon must be performed (Figure 4A); in this group, another option is Bilhaut–Cloquet surgery (Figure 4B) (8, 12–14).

### Statistical analysis

All statistical analyses were performed using the SPSS 22.0 statistics package (SPSS, Chicago, IL, USA). Categorical parameters are expressed as frequencies and percentages. Quantitative data are expressed as the mean ± standard deviation and range. The chi-squared test was used to compare the gender, side, bifurcation level, and dominant thumb in these groups. The tests were two-sided, and a *p*-value of <0.05 was considered significant.

## Results

A total of 35 girls and 57 boys with CTD (*n* = 92 thumbs) met the inclusion criteria. The right side was involved in 58 patients (63%), and the left side was involved in 34 patients (37%). The male-to-female and right-to-left ratios were 1.6:1 and 1.7:1, respectively. The average age at the time of surgery was 29.3 ± 2.8 months (range 11–159).

Among included thumbs, 86 out of 92 could be classified according to the Wassel–Flatt (3) classification as type III, while 92 out of 92 were classified as type C2 according to the Wu et al. classification.

**Figure 5:**
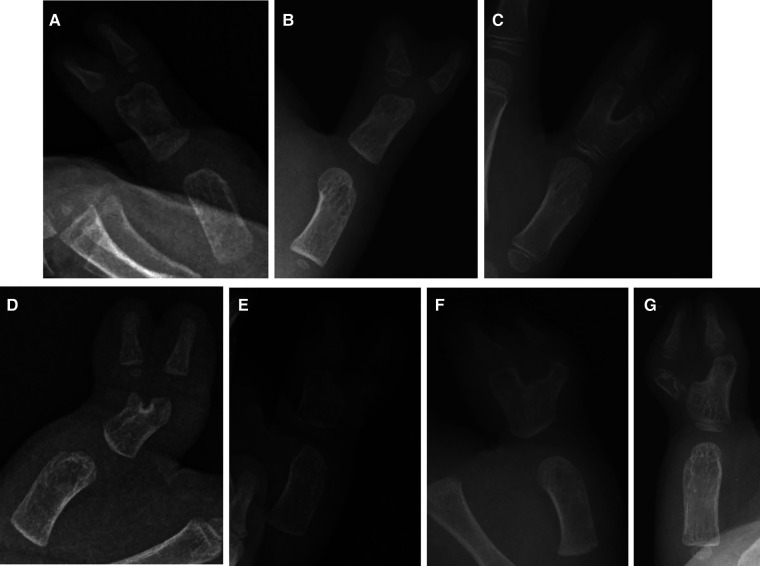
Radiographs of the special pathoanatomy of CTDs in parallel type (**A**), divergent type (**B–E**), and convergent type (**F, G**) groups.

According to the Kim et al. system, 55 (59.8%) CTDs, including two cases of special pathoanatomy that were shown as distal bifurcation incisura of the proximal phalanx, were type 1 (Figure 5A); 24 (26.1%), including 13 cases of special pathoanatomy that were shown as a phalanx slightly widened to accommodate both distal phalanges with an interphalangeal joint deviation of the main thumb but not typical bifurcation, a proximal coaxial bifurcation of the proximal phalanx with a common epiphysis, or a proximal widened basal deformity of the proximal phalanx without typical bifurcation, were type 2 (Figures 5B–E); and 13 (14.1%), including 12 cases of special pathoanatomy that were shown as a proximal widened basal deformity of the proximal bifurcation phalanx with a common epiphysis, were convergent type 3 (Figures 5F,G).

All CTDs (*n* = 92) could be classified according to the proposed system: 76 (82.6%) had no deviation of the IP joint of the main thumb (type I), 10 (10.9%) had an ulnar deviation of the IP joint of the main thumb (type II), and 6 (6.5%) had a radial deviation of the IP joint of the main thumb (type III).

Table 1 shows the gender, laterality, size of hypoplastic duplications, type of surgical procedure, and bifurcation level at the proximal phalanx according to the Kim et al. system and Wu et al. classification (Table 1).

**Table 1:** Kim et al. (2) and Wu et al. (10) systems according to demographic characteristics of patients.

Characteristics *n* (%)		Gender		Laterality		Dominant thumb		Bifurcation level		Total
		Male	Female	Right	Left	Ulnar	Radial	Distal	Proximal	
Kim et al. system	I	33 (60.0)	22 (40.0)	39 (70.9)	16 (29.1)	52 (94.5)	3 (5.5)	40 (72.7)	15 (27.3)	55 (59.8)
	II	16 (66.7)	8 (33.3)	11 (45.8)	13 (54.2)	24 (100)	0 (0)	13 (54.2)	11 (45.8)	24 (26.1)
	III	8 (61.5)	5 (38.5)	8 (61.5)	5 (38.5)	13 (100)	0 (0)	3 (23.1)	10 (76.9)	13 (14.1)
Wu et al. system	A	47 (61.8)	29 (38.2)	50 (65.8)	26 (34.2)	73 (96.1)	3 (3.9)	46 (60.5)	30 (39.5)	76 (82.6)
	B	6 (60.0)	4 (40.0)	5 (50.0)	5 (50.0)	10 (100)	0 (0)	10 (100)	0 (0)	10 (10.9)
	C	4 (66.7)	2 (33.3)	3 (50.0)	3 (50.0)	6 (100)	0 (0)	1 (16.7)	5 (83.3)	6 (6.5)
Total		57 (62.0)	35 (38.0)	58 (63.0)	34 (37.0)	89 (96.7)	3 (3.3)	56 (60.9)	36 (39.1)	92
Chi-squared		0.546		6.103		1.717		23.048		—
*p-*value		0.996		0.293		0.911		0.000		—

Table 2 compares the proportion of IP joint alignment characteristics of the main thumb in patients with CTD according to the Kim et al. classification system (Table 2).

**Table 2:** Alignment of the interphalangeal joint of the main thumb according to the Kim et al. system.

IP alignment Characteristics (Wu et al. classification)		Kim et al. classification			Total
		I	II	III	
Main thumb	No deviation (type A)	55 (100)	15 (62.5)	6 (46.1)	76 (82.6)
	Ulnar deviation (type B)	0 (0)	8 (33.3)	2 (15.4)	10 (10.9)
	Radial deviation (type C)	0 (0)	1 (4.2)	5 (38.5)	6 (6.5)
Total		55 (59.8)	24 (26.1)	13 (14.1)	92
Chi-squared		37.487			
*p-*value		0.000			

## Discussion

Our study found that classifying C2 CTDs into three subtypes (I, II, and III) based on the alignment of the IP joint of the main thumb allows the radiological appearance of these three subtypes to be identified (2, 10). The system is useful for classifying the various proximal phalanx deformities of C2 CTDs and may potentially aid in surgical strategy, but the role of the classification in guiding surgical treatment requires further investigation. In addition, surgical management depends not only on radiographic features but also on thumb development and size, parental choice, and healthcare setting.

Kim et al. introduced a system of three subtypes, 1–3, and found that collateral ligament reconstruction after excision of only the distal phalanx of the supernumerary thumb with preservation of the proximal phalanx of the main thumb is associated with better clinical and radiographic outcomes than excision of the proximal phalanx of the extra thumb (2). However, the subtype system introduced by Kim et al. is not complete regarding the pathoanatomy of proximal phalanx deformities and therefore its use in surgical practice is limited (2).

Our findings showed type C2 CTD is prevalent in boys with a right-sided trend, which is in accordance with most previous reports. In particular, Kim et al. (*n* = 32), Baek et al. (*n* = 5), and Horii et al. (*n* = 11) reported a male-to-female ratio ranging between 1.5–1.7 and 1, while in our series (*n* = 92), the ratio ranged between 1.6 and 1; in addition, these previous studies reported right-to-left ratio between 1.8–4 and 1, while in our series the ratio ranged between 1.7 and 1 (2, 8, 12). These discrepancies may be related to differences in samples, economic status, level of medical care, and environmental factors (2, 8, 10–12).

According to the Kim et al. system, type I CTD was the most common (59.8%), followed by type II (26.1%) and type III (14.1%) (2). Although all CTDs of our series could be classified according to the system of Kim et al., the incidence of the various subtypes we found in our work is different from those reported in the study of Kim et al. (type I: 59.8% vs. 43.75%; type II: 26.1% vs. 43.75%; type III: 14.1% vs. 12.5%); in addition, the number of cases was relatively low and some pathoanatomical characteristics of CTDs may have not been included due to their rarity. There were two (3.6%), 13 (54.2%), and 12 (92.3%) cases of special pathoanatomy in subtypes I, II, and III, respectively (Figure 5). In addition, the Kim et al. subtype system is based solely on the interval between the main and duplicate thumbs and not on the morphological features of the main thumb (2). For example, in the divergent or convergent type, the alignment of the IP of the main thumb may be without deviation, with ulnar deviation, or with radial deviation; this aspect was not considered by Kim et al. Some surgeons do not rely on a subtype system to determine treatment but may instead use a combination of skin, tendon, and skeletal features to guide surgical decisions (13–17).

The classification system we used in this work is based on a radiographic analysis of the alignment of the IP joint of the main thumb and its anatomy. All CTDs could be classified according to this system; in particular, the most frequent subtype was the one with no deviation (type I; 82.6%), followed by the one with ulnar deviation (type II; 10.9%) and the one with radial deviation (type III; 6.5%). It is interesting to note that the duplication of the distal phalanx originates in most cases at the level of its distal part in type I (60.5%) and II (100%) and from its most proximal part in type III (83.3%). This finding is important as IP joint alignment of the main thumb and duplication characteristics can influence the choice of surgical treatment. In patients with type I deformity, the surgical treatment implies the excision of the distal phalanx in association with partial preservation of the proximal phalanx of the extra thumb to maintain the remaining digit in good alignment; alternatively, the proximal phalanx can be completely excised and the thenar muscle reconstructed or a Bilhaut–Cloquet procedure can be performed (Figures 2A–D) (2, 11, 12). Although the Bilhaut–Cloquet procedure allows obtaining a normal-sized thumb with a stable interphalangeal joint, it has limitations, such as the technical difficulty of combining all segments of a duplicated thumb, possible later physeal growth arrest, joint stiffness, and nail-plate deformity (8, 12). We found that the most common type of duplication with thumbs of comparable size could be successfully treated with the Bilhaut–Cloquet procedure: type A in 75% of cases, followed by type C in 16.7% of cases.

In patients with type II deformity, the surgical treatment includes excision of the extra thumb, corrective osteotomy of the proximal phalanx, and ligament/periosteal sleeve reconstruction (Figure 3).

In patients with type III deformity, the surgical treatment must consider the complex pathoanatomy of the deformity, including bony deformity and abnormal location of tendons and thenar muscle (18–20). Surgery includes excision of the extra thumb, corrective osteotomy of the proximal phalanx, a reconstruction of the ligament/periosteal sleeve, and a reconstruction of the thenar muscle or flexor tendon; alternatively, the Bilhaut–Cloquet procedure and soft tissue reconstruction with or without nail bed reconstruction can be performed (one case with similar size of hypoplastic duplications in this study) (Figures 4A,B). Although more research is needed to confirm whether such procedures are feasible for such small thumbs, surgery may be postponed until the thumb is large enough.

It should be noted that certain limitations were observed in the present study: (1) this is a descriptive and retrospective study, but it includes a relatively large cohort of patients with type C2 CTD and all subtypes are likely to be represented; (2) even though they have been used in multiple publications, none of the classification systems used in this study have had their reliability tested; (3) although the proposed classification has some advantages compared to previously published systems, it still has not yet been tested to guide surgical treatment; (4) finally, most patients (89.1%) underwent simple excision and soft tissue reconstruction of the extra thumb, but not exactly the procedure we now propose, such as the Bilhaut–Cloquet procedure and corrective osteotomy of the proximal phalanx. Therefore, further clinical studies with large sample sizes and long follow-ups of patients treated with the different procedures are needed to verify their reliability and feasibility.

## Conclusion

There is space for developing new classification systems, which are fully comprehensive and easy to use in clinical practice. The proposed classification system based on radiographic pathoanatomy complements the system of Wu et al. and can improve communication between professionals. It may potentially aid in surgical strategy, but as our results are preliminary, the role of the classification in guiding surgical treatment in children with type C2 CTD needs to be further investigated.

## Data Availability

The raw data supporting the conclusions of this article will be made available by the authors, without undue reservation.
